# Metformin Protects Cardiomyocyte from Doxorubicin Induced Cytotoxicity through an AMP-Activated Protein Kinase Dependent Signaling Pathway: An *In Vitro* Study

**DOI:** 10.1371/journal.pone.0104888

**Published:** 2014-08-15

**Authors:** Laura C. Kobashigawa, Yan Chun Xu, James F. Padbury, Yi-Tang Tseng, Naohiro Yano

**Affiliations:** Department of Pediatrics, Women & Infants Hospital, The Warren Alpert Medical School of Brown University, Providence, Rhode Island, United States of America; University of Santiago de Compostela School of Medicine – CIMUS, Spain

## Abstract

Doxorubicin (Dox) is one of the most widely used antitumor drugs, but its cumulative cardiotoxicity have been major concerns in cancer therapeutic practice for decades. Recent studies established that metformin (Met), an oral anti-diabetic drug, provides protective effects in Dox-induced cardiotoxicity. Met has been shown to increase fatty acid oxidation, an effect mediated by AMP activated protein kinase (AMPK). Here we delineate the intracellular signaling factors involved in Met mediated protection against Dox-induced cardiotoxicity in the H9c2 cardiomyoblast cell line. Treatment with low dose Met (0.1 mM) increased cell viabilities and Ki-67 expressions while decreasing LDH leakages, ROS generations and [Ca^2+^]_i._ The protective effect was reversed by a co-treatment with compound-C, an AMPK specific inhibitor, or by an over expression of a dominant-negative AMPKα cDNA. Inhibition of PKA with H89 or a suppression of Src kinase by a small hairpin siRNA also abrogated the protective effect of the low dose Met. Whereas, with a higher dose of Met (1.0 mM), the protective effects were abolished regardless of the enhanced AMPK, PKA/CREB1 and Src kinase activity. In high dose Met treated cells, expression of platelet-derived growth factor receptor (PDGFR) was significantly suppressed. Furthermore, the protective effect of low dose Met was totally reversed by co-treatment with AG1296, a PDGFR specific antagonist. These data provide *in vitro* evidence supporting a signaling cascade by which low dose Met exerts protective effects against Dox via sequential involvement of AMPK, PKA/CREB1, Src and PDGFR. Whereas high dose Met reverses the effect by suppressing PDGFR expression.

## Introduction

Doxorubicin (Dox), an anthracycline antibiotic, has been established as an agent against a wide range of cancers [1]. However, the severe cardiotoxicity of Dox is a major factor limiting its use in the treatment of many malignancies [2].

Intensive investigations of Dox-induced cardiotoxicity have been carried out. The different lines of evidence have provided putative mechanisms, but the precise mechanism underlying Dox-induced cardiotoxicity is not fully elucidated. Most studies favor the hypothesis that free radical-induced oxidative stress plays a pivotal role. This is supported by the chemical structure of Dox and its tendency to generate reactive oxygen species (ROS) during drug metabolism [3]–[5]. Recent findings indicate that endothelial nitric oxide synthase (eNOS) reductase domain converts Dox to an unstable semiquinone intermediate that favors ROS generation [5]. Although gaining less attention than ROS has received, a number of studies suggested that Dox-mediated alteration of Ca^2+^ homeostasis is another possible mechanism of cardiotoxicity. Recent studies have demonstrated that Dox-mediated ROS generation induces increase of intracellular Ca^2+^ ([Ca^2+^]_i_), which plays a critical role in damage of cardiomyocytes [6].

Metformin (Met) is an oral biguanide anti-hyperglycemic drug that is widely used for the management of type 2 diabetes mellitus. The therapeutic effects of Met have been attributed to a combination of improved peripheral uptake and utilization of glucose, decreased hepatic glucose output, decreased rate of intestinal absorption of carbohydrate, and enhanced insulin sensitivity [7], [8]. Beyond its glucose lowering effects, Met has been shown to exhibit antioxidant properties in various tissues and acts to decrease lipid peroxidation, an effect that is independent of its effect on insulin sensitivity [9. 10]. Further, Met has been demonstrated to exert cardioprotective effects that could be due to its direct beneficial effects on cellular and mitochondrial function and therefore be independent of its insulin-sensitizing effect [11].

Through its activation of 5′-adenosine monophosphate-activated protein kinase (AMPK), Met reduces the generation of ROS in cultured endothelial cells [12] and in animal models of heart failure [13], [14] and protects cardiomyocytes from oxidative stress induced by H_2_O_2_ or TNFα [14], [15]. However, the specific mechanism by which Met activates AMPK and the corresponding antioxidant effect has not been established. These antioxidant effects suggest that Met could offer a protection against the cardiotoxicity of Dox, although no data are available to support additional benefits of Met in patients being treated with the anthracycline.

The present study was undertaken to delineate signaling pathways by which Met treatment evokes protective effects against the Dox induced cardiotoxicity. For this purpose, we studied Dox-induced *in vitro* toxicity in a fetal rat cardiomyoblast cell line, H9c2, human fetal cardiomyocyte cell line, RL-14 and rat neonatal primary cardiomyocyte. The results of this study provide evidence that the cardioprotective effects of Met are mediated by activation of the AMPK, PKA Src and platelet-derived growth factor receptor (PDGFR). Furthermore, the protective effects are suppressed with high dose Met (1 mM) treatment secondary to reduced cellular PDGF-receptor (PDGFR) expression.

## Materials and Methods

### Reagents and antibodies

Unless otherwise specified, all materials were reagent grade and obtained from Sigma-Aldrich (St. Louis, MO, USA). Anti-Ki67 antibody was obtained from BD Biosciences (San Jose, CA, USA). Alkaline phosphatase (ALP) conjugated horse anti-mouse IgG antibody was obtained from Vector Laboratory (Burlingame, CA, USA). Anti–phosphorylated/total AMPKα, anti-phosphorylated/total acetyl-CoA carboxylase (ACC) and anti-phosphorylated PDGF receptor β (PDGFRβ) antibodies were obtained from Cell Signaling Technology (Danvers, MA, USA). Anti-phosphorylated/total CREB1, c-Src and total PDGFR-β antibodies were obtained from Santa Cruz Biotechnology (Santa Cruz, CA, USA). Anti-phosphorylated tyrosine antibody was obtained from Millipore (Billerica, MA, USA).

### Cell culture

H9c2 rat fetal cardiomyoblasts (ATCC CRL-1446), RL14 human fetal cardiomyocytes (ATCC PTA-1499) and rat neonatal primary cardiomyocytes (Lonza, Allendale, NJ, USA) were grown in DMEM (Invitrogen, Carlsbad, CA, USA) supplemented with 10% (vol/vol) fetal bovine serum in a humidified atmosphere containing 5% CO_2_ at 37°C. Cells were grown to 70% confluence and quietened overnight in serum-free medium before experiments.

### Cell viability assay

Cell viabilities were estimated using CellTiter-Blue Cell Viability Assay (Promega, Fitchburg, WI, USA). Briefly, viable cells retain the ability to reduce resazurin into resorufin, which is highly fluorescent. Nonviable cells rapidly lose metabolic capacity, do not reduce the indicator dye, and thus do not generate a fluorescent signal. Buffered solution containing highly purified resazurin was added to cells growing on 96-well microplates. The spectral properties of the buffer changed upon reduction of resazurin to resorufin. Fluorescence which was emitted from resorufin was measured with maximum excitation and emission spectra of 560 nm and 590 nm, respectively.

### Lactate dehydrogenase release

Lactate dehydrogenase (LDH) is a cellular enzyme released upon membrane damage and a recognized marker of cell damage or death [16]. LDH released into the incubation medium was estimated using an assay kit from Sigma-Aldrich. In brief, LDH reduces nicotinamide adenine dinucleotide, which is then converted a tetrazolium dye to a soluble, colored formazan derivative; this was measured using a micro plate reader (490 nm).

### Reactive Oxygen Species Assay

Cellular ROS was measured using a detection assay kit (Abcam, Cambridge, MA, USA). In brief, 2′,7′-dichlorofluorescein diacetate (DCFDA), a fluorogenic dye that measures hydroxyl, peroxyl and other ROS activity within the cell, was added to the cells growing in the 96-well plates. After diffusion into the cells, DCFDA was deacetylated by cellular esterases to a non-fluorescent compound, which was later oxidized by ROS into 2′,7′-dichlorofluorescin (DCF), a highly fluorescent compound. Fluorescence from the DCF was detected by fluorescence micro plate reader with maximum excitation and emission spectra of 495 nm and 529 nm, respectively.

### Determination of [Ca^2+^]_i_

Levels of [Ca^2+^]_i_ were measured using fluo-4 (Molecular Probe, Eugene, OR, USA), a fluorescent Ca^2+^-indicator dye. Briefly, after removing the growth medium from the cells growing in 96-well microplates, 100 µL of dye loading solution (1× fluo-4 dye with 2.5 mM of probenecid) was added to each well. After incubation for one hour at 37°C, fluorescence from the fluo-4 was detected with a fluorescence micro plate reader with maximum excitation and emission spectra of 494 nm and 516 nm, respectively.

### Immunohistochemistry

H9c2 cells were seeded on poly-L-lysine coated chamber slides. The cells were fixed with 2% formaldehyde and permeabilized by 0.2% TritonX-100, and incubated with a mouse monoclonal anti-Ki67 antibody overnight at 4°C in a humidified chamber. The cells were then incubated with an ALP conjugated anti-mouse IgG (H+L) secondary antibody for 30 min at room temperature. Bound antibody was detected using the ALP substrate kit (Vector Laboratories) and lightly counterstained with veronal acetate buffered 1% methyl green solution, pH 4.0. Permount (Fisher Scientific, Ottawa, Ontario, Canada) was used as the mounting media and sections were cover slipped. The immunohistochemical studies were repeated four times on samples prepared from independent cultures. The labeling index or the proportion of Ki67 positive cells was calculated according to the following formula: 100× (the number of Ki67-positive nuclei/total number of nuclei). Each image was analyzed four times to obtain an average labeling index.

### AMPK activity assay

Cellular AMPK activities were measured using an AMPK kinase assay kit (Cyclex, Ina, Nagano, Japan). Briefly, cell lysate samples were added to plates coated with a substrate-peptide corresponding to surrounding mouse IRS-1 serine 789 (S789), which contains serine residues that can be phosphorylated by AMPK. After washing, anti-phosphorylated mouse IRS-1 S789 monoclonal antibody was added, then the colorimetric reaction was developed by peroxidase conjugated anti-mouse IgG and tetramethylbenzidine substrate (TMB). The absorbance was measured at 450 nm using a micro plate reader.

### Western blotting

Protein levels of the cell lysates for Western blotting (50 µg/lane) were measured as described [17]. GelCode Blue (Thermo Scientific, Waltham, MA, USA) stain of the post transfer gel was used as the loading control for total and phosphorylated PDGFR-β blotting. The results were visualized with Super Signal West Pico chemiluminescent substrate (Thermo Scientific) and analyzed with the UN-SCAN-IT gel software for Windows (Silk Scientific Inc., Orem, UT, USA).

### PKA activity assay

Cellular PKA activity was measured using a PKA kinase activity kit (Enzo Life Sciences, Farmingdale, NY, USA). Briefly, cell lysates to be assayed were added to PKA substrate coated micro plate wells, followed by the addition of ATP to initiate the phosphorylation reaction. After terminating the kinase reaction, a phosphorylated substrate specific antibody was added to the wells. The phosphor-specific antibody was subsequently bound by peroxidase conjugated secondary antibody. The assay was developed with TMB. The absorbance was measured at 450 nm using a micro plate reader.

### Src activity assay

Protein lysates (1 mg) from H9c2 cells were immunoprecipitated with anti-cSrc antibody. Kinase activity was determined by measuring phosphorylation of a specific Src substrate (KVEKIGEGTYGVVYK) using a Src assay kit (Millipore). Briefly, the cSrc immunoprecipitated beads were incubated with a [γ-^32^P]ATP-ATP-Mg^2+^ mix at 30°C for 10 min. A sample without the substrate peptide was included as a background control. Reactions were terminated by adding 40% trichloroacetic acid. After centrifugation the supernatants that include phosphorylated substrate were transferred onto Whatman P81 ion-exchange cellulose chromatography paper circles (GE Healthcare, Little Chalfont, UK). The paper circles were washed five times in 0.75% phosphoric acid and once in acetone, and then counted in a liquid scintillation counter.

### PI3K assay

PI3K activity was determined with *in vitro* immunoprecipitation lipid kinase assay. Briefly, cell lysates (0.5 mg) were immunoprecipitated with anti-phosphorylated tyrosine antibody-coated protein G-sepharose (GE Healthcare), and the beads were resuspend in assay buffer containing 300 µM adenosine to inhibit phosphoinositide 4-kinase (PI4K) activity [18]. L-α-phosphoinositide (Avanti Polar Lipid, Alabaster, AL, USA) was used as the lipid substrate (2 µg/reaction). After incubation, the final extracted reaction mixtures were spotted on to silica gel coated TLC plates (GE Healthcare), and were run in TLC buffer (65% n-propanol and 0.54 M acetic acid). The results were analyzed by phosphorimaging. Densitometric analysis was performed by using the UN-SCAN-IT gel software.

### Stable transfection

Constructs of wild type (WT), dominant-negative (DN) and constitutively active (CA) AMPKα1 in pcDNA3.1 expression vector were generously provided by Prof. David Carling (MRC Clinical Sciences Centre, Imperial College, London, UK). A construct of constitutive active Src (Y529F) in pUSEamp- was purchased from Millipore.

Short hairpin RNA (shRNA) against cSrc was constructed as follows. Two complementary short hairpin siRNA (shRNA) template oligonucleotides, containing 21-nucleotide target sequences of the rat cSrc tyrosine kinase (5′-AAG TAC AAC TTC CAT GGC ACT-3′, GenBank, AC122515.5), were annealed and ligated into the pScilencer 5.1-H1 Retro vector (Invitrogen, Carlsbad, CA, USA).

Stable transfections of these vectors were performed using Lipofectamine 2000 (Invitrogen), according to the manufacturer's instructions. Individual single cells were isolated and selected with G418 (AMPKα1s and active Src transfected cells, 500 µg/ml) or puromycin (shRNA transfected cells, 5 µg/ml). Phenotypes of the transfected cells were evaluated by AMPK and Src activities (Fig. S1, S2).

### Statistical analysis

Statistics of the densitometric analysis were generated from four independent experiments. Statistical evaluations of the other assays (cell viability, LDH leakage, ROS generation, [Ca^2+^]_i_, AMPK activity, PKA activity, and Src activity) were performed from four independent experiments which tested at least 10 samples each time.

Statistical significance of the difference among groups was analyzed by the paired Student's t test or parametric ANOVA and Ryan's multiple comparison test using Microsoft Exel (Microsoft, Redmond, WA, USA) and ANOVA4 on the Web (http://www.hju.ac.jp/~kiriki/anova4/). All data were represented as the mean ± SD of at least four different experiments. A probability of p<0.05 was considered to represent a significant difference.

## Results

### Effects of Met on Dox-induced cardiomyocyte toxicity

**Figure 1:**
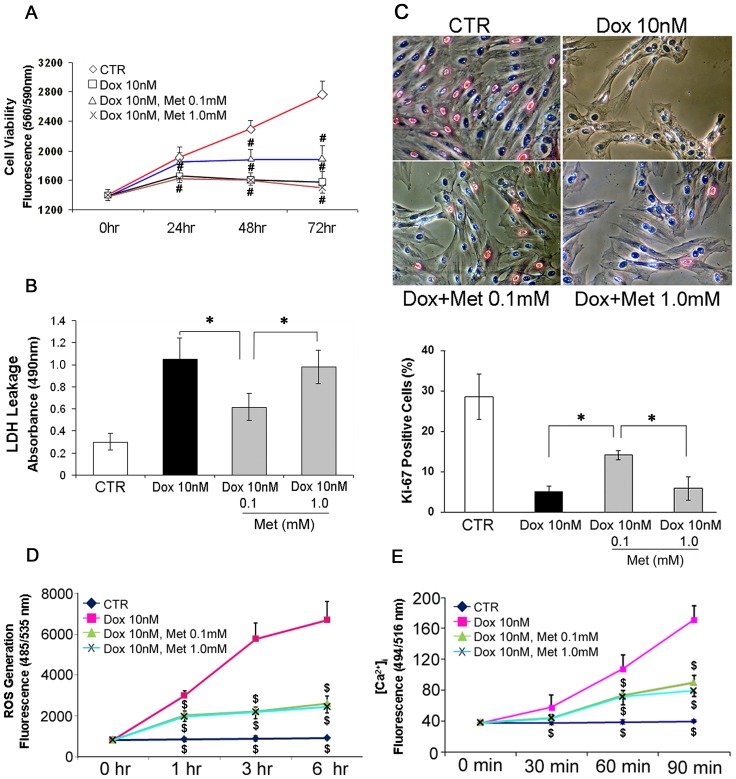
Low dose Met attenuates Dox induced cytotoxicity in H9c2 rat cardiomyoblasts. (A) H9c2 rat immortalized cardiomyoblasts were cultured with reduced FBS (1%) for up to 72 hours in 96-well culture plates under indicated conditions. Viability levels were estimated as described in Materials and Methods. (B) LDH leakages in H9c2 cells after Dox/Met treatment. Cells were treated with indicated conditions for 24 hours in serum free medium. LDH levels in culture supernatants were evaluated using LDH assay kit. (C) H9c2 cells were cultured on poly-L-lysine coated chamber slides for 24 hours under the conditions as indicated in reduced FBS medium. The slides were immunostained with anti-Ki-67 antibody (BD Biosciences) and ALP conjugated secondary antibody (Vector Laboratory). The numbers of Ki-67-positive vessels were counted in six randomized fields of the chambers. The total number of cells from each group was counted and normalized to each chamber. (D) H9c2 cells were cultured with conditions and for hours as indicated in serum free medium. Cellular ROS generations were measured using a detection assay kit. (E) H9c2 cells were cultured with serum free medium under the indicated conditions. Intra-cellular calcium levels ([Ca^2+^]_i_) were measured using fluo-4 (Molecular Probe), a fluorescent Ca^2+^-indicator dye. Values represent mean ± S.D. (n = 4) from quadruplicate samples for each treatment at varying treatment conditions. ^*^ p<0.05; ^#^ p<0.05 vs CTR; ^$^ p<0.05 vs Dox 10 nM alone.

H9c2 cells were seeded in 96-well microplates (3×10^3^ cells/well) and quietened overnight in serum-free medium. In order to minimize the influence of serum on the metabolism of cells while keeping the cells in proliferative status, medium supplemented with reduced (1%, v/v) FBS was used in the experiment [19]. A concentration of Dox (10 nM) was determined to induce up to 40% of growth suppression after 72–96 hours (Fig.S3). The Met concentrations (0.1 and 1.0 mM) used in the experiments were adopted from published *in vitro* studies [20], [21]. Cells were cultured in the reduced serum medium under various combinations of Dox and Met concentrations for up to 72 hours. After treatment with 10 nM of Dox for 72 hours, the cell viability was suppressed to 43.0±5.0% of the vehicle control level. Co-incubation with 0.1 mM of Met reduced the suppression level to 31.1±6.2%. Co-incubation with higher concentration (1.0 mM) of Met, however, did not affect the effect of Dox on cell viability (46.0±3.7% of the control level; Fig. 1A). Dox induced a significant increase in LDH leakage to culture supernatants, another index of cellular damage, after 24 hours of incubation (373.1±115.3% of the control level; Fig. 1B). The increase in LDH leakage was lessened by co-incubation of lower dose of Met (213.6±44.9%) but not by the higher dose of Met (347.8±104.6%). Furthermore, Dox induced a significant reduction of Ki-67 positive cells, (28.6±5.7% vs 5.1±1.4%, CTR vs Dox). The decrease in Ki-67 staining, again, was lessened by co-incubation of lower dose of Met (14.1±1.1%) but not by the higher dose of Met (5.8±2.8%; Fig. 1C). Since Dox-induced cardiotoxicity may be related to cellular ROS generation [3]–[5] or [Ca^2+^]_i_ [6], measurement of these factors could be informative to elucidate the mechanisms of how Met mediates protective effects against Dox-induced cardiotoxicity. Dox treatment significantly increased cellular ROS generation (770.5±154.4% of the control level after 6 hour incubation) and [Ca^2+^]_i_ (437.1±59.9% of the control level after 90 minute incubation). As expected, co-incubation of 0.1 mM of Met partially attenuated the Dox-induced effects (ROS, 295.4±35.6%, [Ca^2+^]_i_, 225.3±31.5%). Unexpectedly, co-incubation with 1.0 mM Met attenuated the effects as well (ROS, 279.8±31.7%, [Ca^2+^]_i_, 198.8±17.1%; Fig. 1D, E). Incubation with Met alone (0.1–1 mM) had no effects on H9c2 cell viability, LDH leakage, ROS generation and [Ca^2+^]_i_ (Fig.S4A-D).

**Figure 2:**
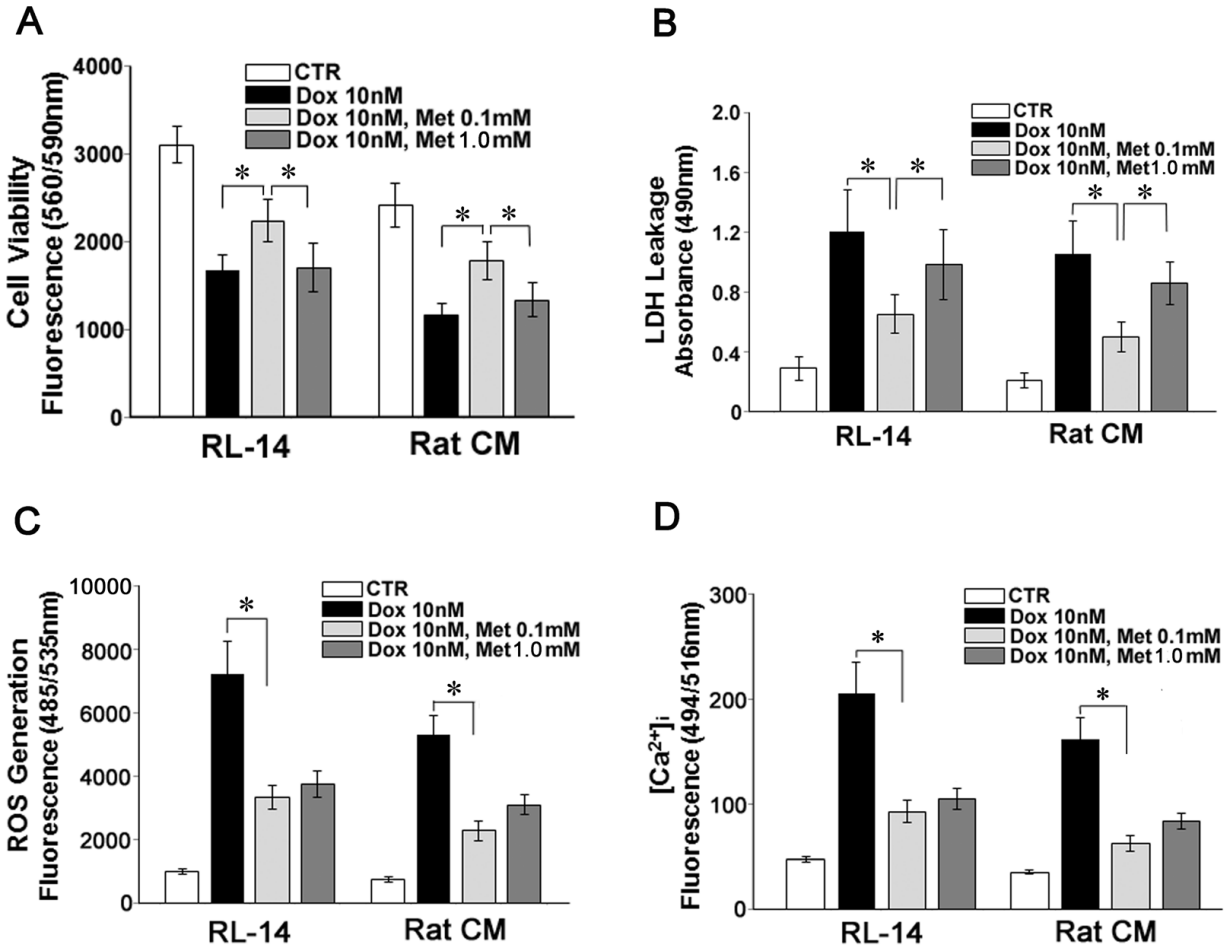
Effects of Met on Dox induced cytotoxicity in RL-14 human cardiomyocytes and rat primary cardiomyocytes. RL-14 human immortalized fetal cardiomyocytes and rat neonatal primary cardiomyocytes (Rat CM) were cultured with same conditions as we did for H9c2 cells, and (A) cell viabilities, (B) LDH leakages, (C) ROS generations and (D) [Ca2+]_i_ were evaluated at 72 hour, 24 hour, 6 hour and 90 minute, respectively. Values represent mean ± S.D. (n = 4) from quadruplicate samples for each treatment at varying treatment conditions. * p<0.05.

In order to confirm the effects of Met on other cardiomyocytes in altered stages of differentiation, cell viability, LDH leakage, ROS generation and [Ca^2+^]_i_ were evaluated using RL14 human fatal cardiomyocytes and rat neonatal primary cardiomyocytes. As shown in Figure 2A-D, Met showed similar effects with these cells.

### Effects of AMPK activity on protection against Dox-induced cardiomyocyte toxicity

**Figure 3:**
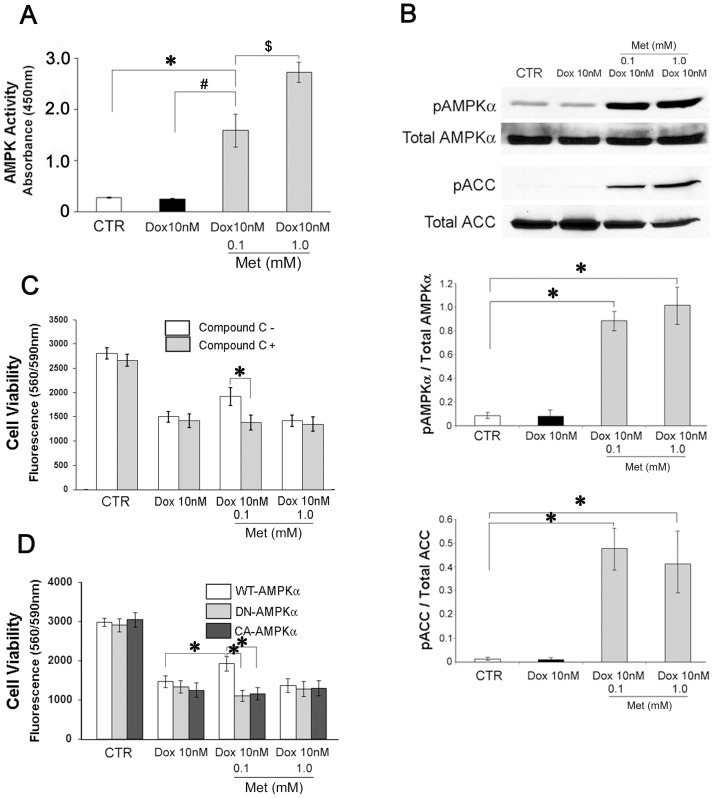
Protective effect of Met against Dox induced cytotoxicity depends on AMPK activity. (A) H9c2 cardiomyoblasts were cultured with reduced FBS (1%) for 72 hours, and then cell lysates were isolated with RIPA buffer supplemented with appropriate phosphatase inhibitors. AMP-activated protein kinase activities in the cell lysates were measured using an AMPK Kinase Assay Kit (Cyclex). (B) H9c2 cell lysates from 72 hour-culture were subject to western blotting using antibodies against phosphorylated or total AMPKα and acetyl-CoA carboxylase (ACC). The histograms show the densitometric scanning results. (C) H9c2 cells were cultured for 72 hours with reduced FBS (1%) in 96-well culture plates under indicated conditions with or without compound-C (10 µM). Cell viabilities were evaluated as described in Materials and Methods. (D) H9c2 cells stably over expressed wild type (WT), dominant negative (DN) or constitutively active (CA) AMPKα cDNA were cultured for 72 hours with reduced FBS (1%) under the indicated conditions. The cell viabilities were evaluated as described. Values represent mean ± S.D. from at least ten (A, C, D) or quadruplicate (B) samples for each treatment at varying treatment conditions. * p<0.05 vs CTR; # p<0.05 Dox 10 nM vs Dox 10 nM + Met 0.1 mM; $ p<0.05 Met 0.1 mM vs Met 1.0 mM (A), * p<0.05 (B, C, D).

Recent advances in the understanding of Met action have centered on the discovery that Met leads to increased phosphorylation and activation of AMPKα [22], [23]. Therefore, the relationship between the protection against Dox-induced cardiomyocyte toxicity and AMPK activity were examined in this study. Co-incubation of Met (0.1 and 1.0 mM) with 10 nM Dox significantly enhanced cellular AMPK activity in H9c2 cells after 72 hour incubation (0.1 mM Met; 584.0±88.3%, 1.0 mM Met; 1009.3±127.2% of the control level), while incubation with Dox alone showed no effect (91.2±1.6%; Fig.3A). AMPK phosphorylates and inhibits acetyl-CoA carboxylase (ACC), the key enzyme that controls generation of malonyl-CoA from acetyl-CoA. As malonyl-CoA decreases fatty acid oxidation through inhibition of carnitine palmitoyl transferase-1 (CPT-1), phosphorylation of ACC relieves the inhibition of CPT-1, favoring fatty acid oxidation. In the current study, Met increased AMPK (0.1 mM; 1053.4±134.6%, 1.0 mM; 1224.3±299.2% of the control level) and ACC (0.1 mM; 4019.5±830.7%, 1.0 mM; 3501.5±1238.9% of the control level) phosphorylation in H9c2 cells after 72 hours of incubation (Fig. 3B). In order to verify the effects of AMPK activity on the Met induced effects, cells were co-incubated with of compound-C, an AMPK inhibitor. A concentration of the compound-C (10 µM) was determined that did not affect the cell viability while higher concentrations (20 µM, 40 µM) significantly reduce cell viability at 72-hour (Fig. S5). As shown in Fig. 3C, co-incubation of compound-C completely reversed the effects of Met in attenuating Dox-induced reduction in cell viability (compound C-; 68.4±6.9%, compound C+; 51.9±5.6% of the control level). Furthermore, H9c2 cells were stably transfected with plasmids with wild type (WT), dominant-negative (DN) or constitutively active (CA) AMPKα1 cDNAs (Fig. S1). In DN-AMPKα cells the protective effect of 0.1 mM Met was completely abrogated (Fig. 3D). These findings suggested that the 0.1 mM Met-mediated protective effects were dependent on the AMPK activity. Some clones of the CA-AMPKα1 transfected H9c2 cells showed extremely high AMPK activities which were comparable to those of cells treated with 1.0 mM of Met (Fig. S1). Interestingly, the CA-AMPKα1 transfected cells which obtained extremely high AMPK activities did not show protective effects against Dox-induced toxicity (Fig. 3D, Fig. S6). These results suggest that Met protected cardiomyocytes through moderately enhanced AMPK activities and the protective effect was reversed if the AMPK activity exceeded a certain threshold.

### Effects of PKA activity on protection against Dox-induced cardiomyocyte toxicity

**Figure 4:**
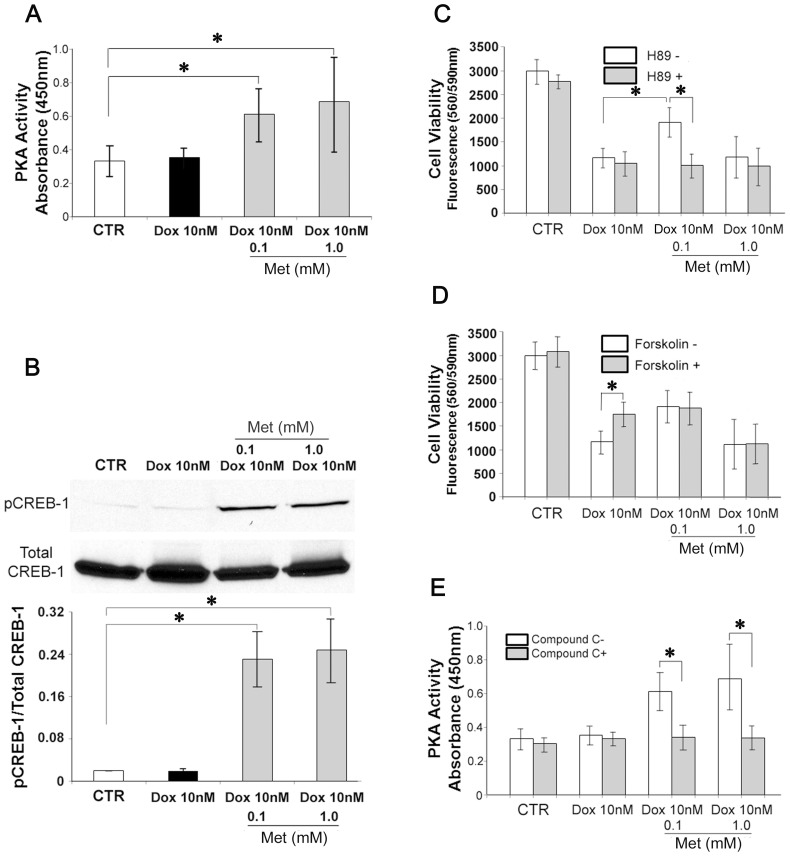
Protective effect of Met against Dox induced cytotoxicity is mediated by cAMP-PKA-CREB1 signaling pathway. (A) H9c2 cardiomyoblasts were cultured with reduced FBS (1%) for 72 hours, with indicated conditions and then cell lysates were isolated with RIPA buffer supplemented with appropriate phosphatase inhibitors. Protein kinase-A (PKA) activities in the cell lysates were measured as described in Materials and Methods. (B) H9c2 cell lysates from the 72 hour-culture were subject to Western blotting using antibodies against phosphorylated or total cAMP response element-binding protein-1 (CREB-1). The histograms show the densitometric scanning results. (C) H9c2 cells were cultured for 72 hours with reduced FBS (1%) under indicated conditions with or without H-89 (10 µM). The cell viabilities were evaluated as described. (D) H9c2 cells were cultured for 72 hours with reduced FBS (1%) for hours under indicated conditions with or without forskolin (1 µM). The cell viabilities were evaluated as described. (E) H9c2 cells were cultured for 72 hours with reduced FBS (1%), under indicated conditions with or without compound-C (10 µM) and then the cell lysates were evaluated for PKA activities. Values represent mean ± S.D. (n = 4) from quadruplicate samples for each treatment at varying treatment conditions. *, Statistically significant (p<0.05).

In previous studies, we described PKA as a crucial factor in cell survival of isoproterenol stimulated H9c2 cells [24], [25]. To determine the involvement of PKA in the Met mediated protective effects, PKA activities were measured by *in vitro* kinase assay. In the cells co-incubated with Met, PKA activities were significantly elevated (Met 0.1 mM; 187.6±37.6%, Met 1.0 mM; 211.6±81.5% of the control level; Fig. 4A). Dox treatment alone had no effect on either PKA activity (108.2%±23.9% of the control level) or phosphorylation of CREB-1 (102.5±26.3% of the control level), a downstream transcription factor of the cAMP/PKA signaling pathway. Met also significantly increased CREB-1 phosphorylation (Met 0.1 mM; 1209.1±294.0%, Met 1.0 mM; 1298.1±439.3% of the control level; Fig. 4B). Inhibition of PKA activity with H89 (10 µM) abolished the protective effect of 0.1 mM Met (Met 0.1 mM, H89-; 64.5±8.4%, H89+; 36.5±4.0% cell viability of the control level; Fig. 4C). Forskolin (1 µM), an activator of adenylyl cyclase, attenuated Dox-induced toxicity on the cardiomyocyte (forskolin-; 39.6±6.9%, forskolin+; 57.5±8.5% cell viability of the control level), but did not enhance the effect of 0.1 mM Met on cell viability (forskolin-; 64.5±8.4, forskolin+; 61.7±9.0 cell viability of the control level; Fig.4D). Furthermore, the Met induced PKA activities were reversed by co-incubation with compound-C (compound C-; 187.6±37.6%, compound C+; 112.7±19.1% of the control level; Fig. 4E). These data suggested that the protective effect of Met was mediated by the PKA activation, and the PKA activation was dependent on the AMPK activity.

### Met-mediated protective effect is dependent on Src family tyrosine kinase

**Figure 5:**
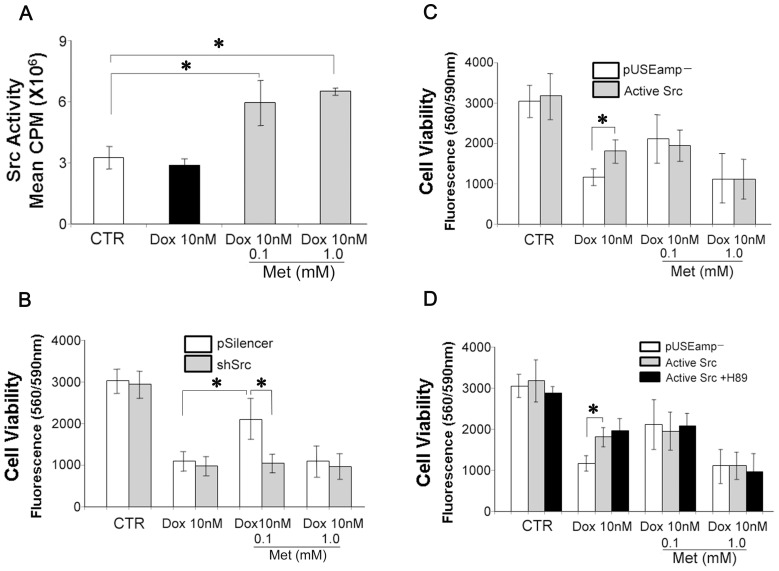
Protective effect of Met against Dox induced cytotoxicity is mediated by PKA dependent Src activation. (A) H9c2 cardiomyoblasts were cultured with reduced FBS (1%) for 72 hours, with indicated conditions. Src kinase activity was determined as described in Materials and Methods. The histograms show the average Src activity in CPM. (B) H9c2 cells stably transfected with short hairpin RNAi against cSrc (shSrc) or scrambled oligo encoded control plasmid (pSilencer) were cultured for 72 hours with reduced FBS (1%) the indicated conditions. The cell viabilities were evaluated as described. (C) H9c2 cells stably transfected with a constitutively active Src or a control empty vector (pUSEamp-) were cultured for 72 hours with reduced FBS (1%) under the indicated conditions. The cell viabilities were evaluated as described. (D) H9c2 cells stably transfected with an active Src or a control plasmid (pUSEamp-) were cultured for 72 hours under the indicated conditions with or without H89 (10 µM). Values represent mean ± S.D. (n = 4) from quadruplicate samples for each treatment at varying treatment conditions. *, Statistically significant (p<0.05).

We have shown that the Src-family tyrosine kinase is involved in βAR-mediated anti-apoptosis in H9c2 cells [25]. In the present study, to explore the role of Src in Met-mediated cell protection, a series of experiments were initiated. First, cells were cultured with vehicle, Dox alone, or Dox in combination with Met for 72 hours, and then the cellular Src activity was measured. As shown in Fig. 5A, Met induced Src activation both in 0.1 and 1.0 mM of concentrations (189.9±51.9% and 203.3±42.0% of the control level, respectively).

Second, cSrc was knocked down by shRNA transfection. We have reported effective knockdown of Src at the RNA and protein levels in H9c2 cardiomyocyte with this approach [24], [25]. In this study, H9c2 cells were stably transfected with a scrambled oligo control vector (pSilencer) or shSrc. Knock down of Src effectively obliterated the protective effect of 0.1 mM Met on the Dox-induced toxicity (pSilencer; 69.0±6.0%, shSrc; 35.7±6.0% cell viability of the control level; Fig. 5B).

Third, constitutively active Src cDNA (Y529F) or a control vector (pUSEamp-) was stably transfected into H9c2 cells. Overexpression of the constitutively active Src significantly reduced Dox-induced cytotoxicity (pUSEamp-; 38.9±8.3%, active Src; 58.5±11.9% cell viability of the control level; Fig. 5C). The protective effect of 0.1 mM Met, however, was not further increased by overexpression of the constitutively active Src (pUSEamp-; 69.1±9.0%, active Src; 63.7±18.1% cell viability of the control level). Moreover, inhibition of PKA activity with H89 in these Src overexpressing cells did not abrogate the protective effect of 0.1 mM Met (active Src; 63.7±18.1%, active Src +H89; 72.6±10.1% cell viability of the control level; Fig.5D), which was observed in non-transfected H9c2 cells (Fig. 5C), suggesting PKA acts upstream of Src in this signaling pathway. These observations suggest that Src is a critical factor in the Met-induced anti-cytotoxic effect by functioning downstream of AMPK/PKA signaling pathway.

### Expression of PDGFR is down regulated by co-incubation with 1.0 mM Met

**Figure 6:**
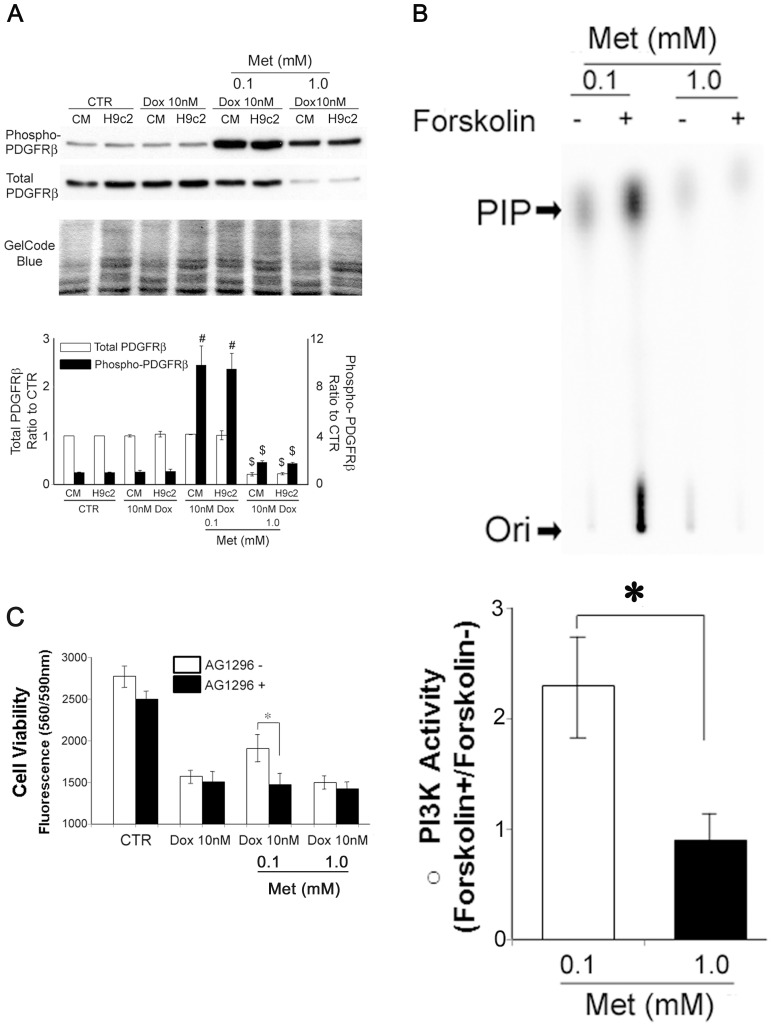
High dose Met abrogates the protective effects against Dox by suppressing the PDGFR expression. (A) Rat neonatal primary cardiomyocytes (CM) and H9c2 cardiomyoblasts were cultured with reduced FBS (1%) for 72 hours, with the indicated conditions. Cell lysates from the culture were subject to Western blotting using antibodies against phosphorylated or total platelet-derived growth factor receptor β-subunit (PDGFRβ). An image of the gel stained with GelCode Blue dye (Thermo) after transfer was shown as a loading monitor. The histograms show the densitometric scanning results. (B) Quiescent H9c2 cells were incubated with 0.1 or 1.0 mM metformin (Met) for 5 minutes with or without forskolin (1 µM). After the treatment, PI3K activities were determined as described in Materials and Methods. The histograms show the densitmetric scanning results. PIP, phosphoinositide 3-phosphate. Ori, origin of migration in thin-layer chromatography. (C) H9c2 cells were cultured for 72 hours with reduced FBS (1%), under the indicated conditions with or without AG1296 (10 µM). The cell viabilities were evaluated as described. * p<0.05, # p<0.05 vs CTR, $ p<0.05 vs Dox 10 nM + Met 0.1 mM. Values represent mean ± S.D. (n = 4) from quadruplicate samples for each treatment at varying treatment conditions.

We have shown that PDGFR plays a pivotal role in survival of H9c2 cells [24], [25]. In order to explore the roles of PDGFR signaling in Met-induced survival in Dox-treated H9c2 cells, experiments were performed as follows. Cellular expression and phosphorylation levels of PDGFR were evaluated by western blotting. As shown in Fig. 6A, the phosphorylation levels of PDGFRβ was increased in 0.1 mM Met treated rat neonatal primary cardiomyocytes and H9c2 cells. In contrast, the expression levels of the receptor were significantly suppressed in 1.0 mM Met treated cells. The PDGFR expression was also downregulated in the CA-AMPK transfected H9c2 cells which showed extremely high AMPK activity (Fig. S7). With the hypothesis that an AMPK/PKA/Src/PDGF sequence was a critical pathway for the Met induced cardiomyocyte protection and to verify the functional insufficiency of PDGFR response against PKA stimulation in 1.0 mM Met treated cells. We performed an experiment using activities of phosphoinositide 3-kinase (PI3K), a downstream molecule of PDGFR signaling, as an index for PDGFR sensitivities against forskolin. In 0.1 mM Met treated cells, forskolin stimulation induced a significant increase in PI3K activity while 1.0 mM Met treated cells showed no effect (Fig. 6B). Furthermore, co-incubation with AG1296, a PDGFR specific antagonist, abrogated 0.1 mM Met induced protective effect against Dox-mediated cytotoxicity in H9c2 cardiomyocytes (Fig. 6C). These findings suggested that PDGFR signaling is a crucial factor in Met-induced protective effect against Dox-mediated cytotoxicity of H9c2 cells. The reversal of the effect with the higher concentration (10 mM) of Met is a result of the down regulation and the functional loss of PDGFR.

## Discussion

In this study, we demonstrated that metformin (Met), an anti-diabetic agent, has a clearly protective effect against doxorubicin-induced toxicity on cardiomyocytes through activation of AMPK. Whether Met, as a consequence of its modulated metabolism, influences cardiomyocyte survival remains unknown. We have previously reported a pro-survival/proliferation pathway in cardiomyocyte [24], [25] and renal mesangial cells [26], [27] involving G-protein coupled receptor (GPCR)-PKA-Src-receptor tyrosine kinase. The present study provides evidences that AMPK and PKA are activated sequentially following Met treatment. However, the mechanisms of AMPK dependent PKA activation are not fully clarified. A signaling interaction between AMPK and PKA was described in a hypothalamic cell line [28] and AMPK mediated CREB, a downstream transcription factor of a cAMP/PKA signaling pathway, activations were reported in hepatocyte [29] and neuronal cells [30]. Met/AMPK-induced transactivations of several GPCRs were also described in a pancreatic β-cell line [31]. All of the findings in the previous publications suggest high probability for the existence of AMPK/PKA/CREB or AMPK/GPCR/PKA/CREB signaling cascades in some cells.

Src has been identified as a key effector of PKA signaling [32], [33]. In this study, we showed a pivotal role of the Src kinase as we have previously reported [24]–[26]. We have demonstrated that GPCR/PKA/Src to receptor tyrosine kinase (RTK) link is a notable pro-survival signaling pathway in cardiomyocytes and renal mesangial cells. In the present study, we provide convincing evidence that AMPK activation is critical in Met-mediated resistance against the Dox-induced cytotoxicity and that this protective effect was accomplished via sequential activation of PKA/Src/PDGFR.

A very novel and interesting finding in this study is the dual effects of Met on cardiomyocyte survival. We showed that, at lower concentrations (0.1 mM), Met protected cardiomyocytes from the Dox-induced toxicity, whereas a higher concentration (1.0 mM) of Met failed to do so despite the fact that higher concentration of Met induced increases in many parameters we measured including AMPK. PKA, CREB1 and Src with even more potent manners than those with a lower concentration of Met. Moreover and most notably, a higher concentration of Met showed similar effects on ROS generations and [Ca^2+^]_i_ in Dox intoxicated cardiomyocytes. Our data suggested that the biphasic effect was caused by dose dependent alteration in PDGFR expression. Excessive AMPK activity in 1.0 mM Met treated cells may induce the suppression of the PDGFR expression. And the attenuated PDGFR signaling may be a factor to wipe out the Met induced effects of decreased ROS generation and [Ca^2+^]_i_ against Dox treatment. In the meanwhile, the elevated PDGFR activity in cells treated with the lower concentration of Met may overcome the Dox induced cardiotoxicity to maintain cell viabilities. However, this hypothesis is still premature because of lack of the bibliographical evidences to support it. Further investigations should be addressed to provide a logical explanation for these unexpected findings.

PDGF was originally identified in serum and platelets as a strong mitogen for fibroblasts, smooth muscle cells, and glial cells [34]. PDGF signaling plays important roles in the pathogenesis of several proliferative and degenerative diseases such as tumorigenesis, arteriosclerosis, and fibrosis [35]. In the present study, we demonstrated that the higher dose of Met resulted in a significant reduction of the levels of PDGFR. In contrast, lower dose of Met did not reduce the levels of PDGFR but enhanced the cellular activity of the receptor tyrosine kinase. More important, the protective effect of lower dose of Met is abrogated by PDGFR antagonist; clearly PDGFR signaling is important for the dual effect of Met-mediated protection against Dox toxicity.

In the last few years, several studies concerning about the protective effects of Met against the Dox toxicity has been published elsewhere [36]–[38]. Interestingly, in these papers, they demonstrated that even a dose of 4 mM of Met were able to protect cardiomyocytes in culture from the cytotoxic effect of doxorubicin. The cause of the discrepancy between our and their findings is not elucidated at present. Supposedly, differences in the lineage of the cells used (H9c2 vs HL-1) and dosage of Dox applied (10 nM vs 5 µM) may deduce to the inconsistency. Elucidating details in this discrepant action of Met on cardiomyocyte will benefit further understanding a mechanism of the protective effect of Met against cardiotoxicity of Dox.

The major proteolytic pathway involving the ubiquitin-proteasome system (UPS) is dependent on ATP [39]. Activation of AMPK results in the stimulation of a variety of cellular processes involved in the production of ATP, e. g., glucose uptake [40], protein synthesis [41] and UPS-mediated protein degradation [42]. In the present study, AMPK activity in 1.0 mM Met treated cells was significantly higher than those in 0.1 mM Met treated cells (Fig. 3A). Furthermore, constitutively active AMPKα cDNA transfected cells, which had even higher AMPK activities than those of 1.0 mM Met treated cells, showed suppressed PDGFR expression (Fig. S1, S6). Considering these findings, AMPK activities beyond a certain threshold may promote PDGFR degradation in H9c2 cardiomyocytes. Elucidating further details in this effect on PDGFR expression should be addressed in the future.

**Figure 7:**
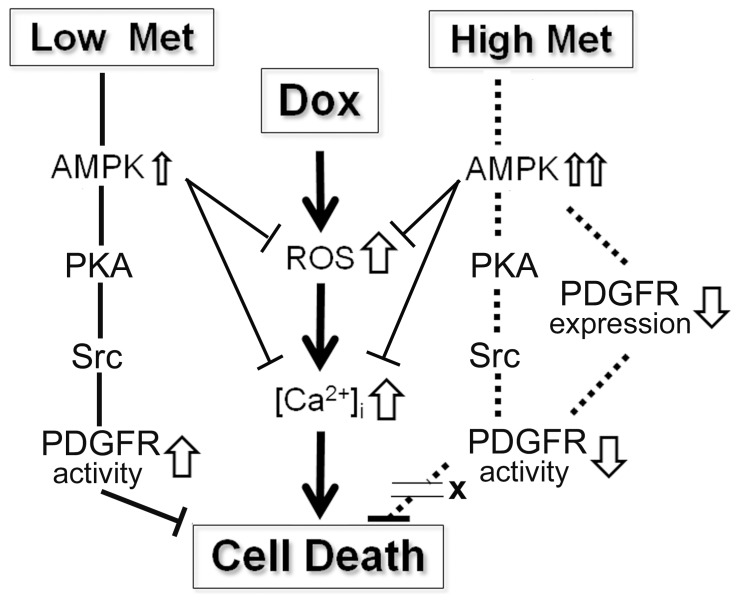
A hypothetical pathway for Met-mediated protection against Dox-induced toxicity in cardiomyocytes. Low dose (0.1 mM) Met induces moderate AMPK activation which is followed by sequential activations of PKA, Src and PDGFR and results in protection of the cells from Dox toxicity. High dose (1.0 mM) Met also leads to the sequential activations of AMPK, PKA and Src. With both dosages of the Met treatment, Dox induced ROS generations and [Ca^2+^]_i_ levels are suppressed. However, the excessive activities of AMPK with the high dose Met causes a suppression of PDGFR expression in cardiomyocytes and the protective effect of low dose Met is abrogated.

We have investigated the roles of AMPK and PKA as crucial factors in Met induced resistance against Dox toxicity on H9c2 cardiomyocytes. We demonstrated that PDGFR transactivation is involved in this pathway. We further established that Src played a pivotal role in the signaling pathway by functioning between PKA and PDGFR. We also described cellular PDGFR expression levels as regulatory factor for the protective effect of Met on cardiomyocytes. Based on these findings, despite the fact that bibliographic references to support this hypothesis are limited at present, we propose a hypothetical pathway for the Met-mediated protective effect against the Dox-induced toxicity on cardiomyocyte (Fig. 7). Although there are other components needed to be identified in this signaling pathway, our findings nonetheless provide important information for the protective effects of Met which has attracted attention recently. Elucidating further details in this signaling pathway should lead to better understanding over the conventional chemotherapy for malignant neoplasms.

## Supporting Information

Figure S1 **AMPK activities in empty vector (pcDNA3), wild type (WT), dominant negative (DN) or constitutively active (CA)-AMPKα transfected H9c2 cardiomyoblasts were as described in Materials and Methods.** Three clones from each transfection were tested. Values represent mean ± S.D. (n = 4) from quadruplicate samples for each treatment. *, Significantly different from control (pcDNA3) (p<0.05).
(TIF)

Figure S2 **Src activities in H9c2 cells stably transfected with empty control vector, shRNA against cSrc (sh-cSrc) or constitutively active Src cDNA H9c2 cardiomyocytes were evaluated as describes in Materials and Methods.** Values represent mean ± S.D. (n = 4) from quadruplicate samples for each treatment. *, Significantly different from control (pSilencer for sh-cSrc and pUSEamp- for active Src) (p<0.05).
(TIF)

Figure S3 **H9c2 cells were cultured for up to 96 hours with reduced FBS (1 with or without the indicated concentrations of Dox.** Cell viabilities were evaluated as described. Values represent mean (n = 4) from quadruplicate samples for each treatment.
(TIF)

Figure S4 **H9c2 cells were cultured for indicated durations with reduced FBS (1%) with or without the indicated concentrations of Met. Cell viabilities (A), LDA leakages (B), ROS generations (C) and [Ca^2+^]_i_ (D) were evaluated as described.** Values represent mean (n = 4) from quadruplicate samples for each treatment.
(TIF)

Figure S5 **H9c2 cells were cultured for up to 72 hours with reduced FBS (1%) with or without the indicated concentrations of compound-C.** Cell viabilities were evaluated as described. Values represent mean ± S.D. (n = 4) from quadruplicate samples for each treatment. *, Significantly different from control (CTR) (p<0.05).
(TIF)

Figure S6 **H9c2 cells which were stably transfected with the indicated plasmids were cultured for 72 hours with reduced FBS (1%) with 10 nM Dox and 0.1 mM of Met.** Cell viabilities and AMPK activities were evaluated as described.
(TIF)

Figure S7 **Cell lysates from quiescent AMPKα plasmids transfected H9c2 cells were subject to Western blotting using antibodies against platelet-derived growth factor receptor β-subunit (PDGFRβ).** An image of the gel stained after transfer was shown as a loading monitor. The histogram shows the densitometric scanning results. *, Significantly different from control (pcDNA3) (p<0.05).
(TIF)

## References

[1] Wang JJ, Cortes E, Sinks LF, Holland JF (1971) Therapeutic effect and toxicity of adriamycin in patients with neoplastic disease. Cancer 28: 837–843. 4329505 10.1002/1097-0142(1971)28:4<837::aid-cncr2820280406>3.0.co;2-4

[2] Gottlieb JA, Gutterman JU, McCredie KB, Rodriguez V, Frei E III (1973) Chemotherapy of malignant lymphoma with adriamycin. Cancer Res 33: 3024–3028. 4748450

[3] Iarussi D, Indolfi P, Casale F, Coppolino P, Tedesco MA, et al (2001) Recent advances in the prevention of anthracycline cardiotoxicity in childhood. Curr Med Chem 8: 1649–1660. 11562284 10.2174/0929867013371888

[4] Wallace KB (2003) Doxorubicin-induced cardiac mitochondrionopathy. Pharmacol Toxicol 93: 105–115. 12969434 10.1034/j.1600-0773.2003.930301.x

[5] Neilan TG, Blake SL, Ichinose F, Raher MJ, Buys ES, et al (2007) Disruption of nitric oxide synthase 3 protects against the cardiac injury, dysfunction, and mortality induced by doxorubicin. Circulation 116: 506–514. 17638931 10.1161/CIRCULATIONAHA.106.652339

[6] Kalivendi SV, Kotamraju S, Zhao H, Joseph J, Kalyanaraman B (2001) Doxorubicin-induced apoptosis is associated with increased transcription of endothelial nitric-oxide synthase. Effect of antiapoptotic antioxidants and calcium. J Biol Chem 276: 47266–47276. 11579094 10.1074/jbc.M106829200

[7] Klip A, Leiter LA (1990) Cellular mechanism of action of metformin. Diabetes Care 13: 696–704. 2162756 10.2337/diacare.13.6.696

[8] Cusi K, Consoli A, DeFronzo RA (1996) Metabolic effects of metformin on glucose and lactate metabolism in noninsulin-dependent diabetes mellitus. J Clin Endocrinol Metab 81: 4059–4067. 8923861 10.1210/jcem.81.11.8923861

[9] Faure P, Rossini E, Wiernsperger N, Richard MJ, Favier A, et al (1999) An insulin sensitizer improves the free radical defense system potential and insulin sensitivity in high fructose-fed rats. Diabetes 48: 353–357. 10334313 10.2337/diabetes.48.2.353

[10] Kanigur-Sultuybek G, Guven M, Onaran I, Tezcan V, Cenani A, et al (1995) The effect of metformin on insulin receptors and lipid peroxidation in alloxan and streptozotocin induced diabetes. J Basic Clin Physiol Pharmacol 6: 271–280. 8852272 10.1515/jbcpp.1995.6.3-4.271

[11] Bhamra GS, Hausenloy DJ, Davidson SM, Carr RD, Paiva M, et al (2008) Metformin protects the ischemic heart by the Akt-mediated inhibition of mitochondrial permeability transition pore opening. Basic Res Cardiol 103: 274–284. 18080084 10.1007/s00395-007-0691-y

[12] Mahrouf M, Ouslimani N, Peynet J, Djelidi R, Couturier M, et al (2006) Metformin reduces angiotensin-mediated intracellular production of reactive oxygen species in endothelial cells through the inhibition of protein kinase C. Biochem Pharmacol 72: 176–183. 16730666 10.1016/j.bcp.2006.04.027

[13] Gundewar S, Calvert JW, Jha S, Toedt-Pingel I, Ji SY, et al (2009) Activation of AMP-activated protein kinase by metformin improves left ventricular function and survival in heart failure. Circ Res 104: 403–411. 19096023 10.1161/CIRCRESAHA.108.190918 PMC2709761

[14] Sasaki H, Asanuma H, Fujita M, Takahama H, Wakeno M, et al (2009) Metformin prevents progression of heart failure in dogs: role of AMP-activated protein kinase. Circulation 119: 2568–2577. 19414638 10.1161/CIRCULATIONAHA.108.798561

[15] Kewalramani G, Puthanveetil P, Wang F, Kim MS, Deppe S, et al (2009) AMP-activated protein kinase confers protection against TNF-α-induced cardiac cell death. Cardiovasc Res 84: 42–53. 19477967 10.1093/cvr/cvp166

[16] Das A, Xi L, Kukreja RC (2005) Phosphodiesterase-5 inhibitor sildenafil preconditions adult cardiac myocytes against necrosis and apoptosis. Essential role of nitric oxide signaling. J Biol Chem 280: 12944–12955. 15668244 10.1074/jbc.M404706200

[17] Tseng YT, Yano N, Rojan A, Stabila JP, McGonnigal BG, et al (2005) Ontogeny of phosphoinositide 3-kinase signaling in developing heart: effect of acute β-adrenergic stimulation. Am J Physiol Heart Circ Physiol 289: H1834–H1842. 16006545 10.1152/ajpheart.00435.2005

[18] Wong K, Cantley LC (1994) Cloning and characterization of a human phosphatidylinositol 4-kinase. J Biol Chem 269: 28878–28884. 7961848

[19] Kalka D, Hoyer S (1998) Long-term cultivation of a neuroblastoma cell line in medium with reduced serum content as a model system for neuronal aging? Arch Gerontol Geriatr 27: 251–268. 18653168 10.1016/s0167-4943(98)00122-8

[20] An D, Kewalramani G, Chan JK, Qi D, Ghosh S, et al (2006) Metformin influences cardiomyocyte cell death by pathways that are dependent and independent of caspase-3. Diabetologia 49: 2174–2184. 16868748 10.1007/s00125-006-0338-9

[21] Yang J, Holman GD (2006) Long-term metformin treatment stimulates cardiomyocyte glucose transport through an AMP-activated protein kinase-dependent reduction in GLUT4 endocytosis. Endocrinology 147: 2728–2736. 16513829 10.1210/en.2005-1433

[22] Zhou G, Myers R, Li Y, Chen Y, Shen X, et al (2001) Role of AMP-activated protein kinase in mechanism of metformin action. J Clin Invest 108: 1167–1174. 11602624 10.1172/JCI13505 PMC209533

[23] Fryer LG, Parbu-Patel A, Carling D (2002) The Anti-diabetic drugs rosiglitazone and metformin stimulate AMP-activated protein kinase through distinct signaling pathways. J Biol Chem 277: 25226–25232. 11994296 10.1074/jbc.M202489200

[24] Yano N, Ianus V, Zhao TC, Tseng A, Padbury JF, et al (2007) A novel signaling pathway for β-adrenergic receptor-mediated activation of phosphoinositide 3-kinase in H9c2 cardiomyocytes. Am J Physiol Heart Circ Physiol 293: H385–H393. 17369456 10.1152/ajpheart.01318.2006

[25] Yano N, Suzuki D, Endoh M, Tseng A, Stabila JP, et al (2008) β-adrenergic receptor mediated protection against doxorubicin-induced apoptosis in cardiomyocytes: the impact of high ambient glucose. Endocrinology 149: 6449–6461. 18719028 10.1210/en.2008-0292 PMC2613054

[26] Yano N, Suzuki D, Endoh M, Zhao TC, Padbury JF, et al (2007) A novel phosphoinositide 3-kinase-dependent pathway for angiotensin II/AT-1 receptor-mediated induction of collagen synthesis in MES-13 mesangial cells. J Biol Chem 282: 18819–18830. 17493931 10.1074/jbc.M610537200

[27] Yano N, Suzuki D, Endoh M, Cao TN, Dahdah JR, et al (2009) High ambient glucose induces angiotensin-independent AT-1 receptor activation, leading to increases in proliferation and extracellular matrix accumulation in MES-13 mesangial cells. Biochem J 423: 129–143. 19604148 10.1042/BJ20082277

[28] Damm E, Buech TR, Gudermann T, Breit A (2012) Melanocortin-induced PKA activation inhibits AMPK activity via ERK-1/2 and LKB-1 in hypothalamic GT1-7 cells. Mol Endocrinol 26: 643–654. 22361823 10.1210/me.2011-1218 PMC5417139

[29] Yuan HD, Piao GC (2011) An active part of Artemisia sacrorum Ledeb. suppresses gluconeogenesis through AMPK mediated GSK3β and CREB phosphorylation in human HepG2 cells. Biosci Biotechnol Biochem 75: 1079–1084. 21670525 10.1271/bbb.100881

[30] Choi IY, Ju C, Anthony Jalin AM, Lee d I, Prather PL, et al (2013) Activation of cannabinoid CB2 receptor-mediated AMPK/CREB pathway reduces cerebral ischemic injury. Am J Pathol 182: 928–939. 23414569 10.1016/j.ajpath.2012.11.024

[31] Pan QR, Li WH, Wang H, Sun Q, Xiao XH, et al (2009) Glucose, metformin, and AICAR regulate the expression of G protein-coupled receptor members in INS-1 β cell. Horm Metab Res 41: 799–804. 19672815 10.1055/s-0029-1234043

[32] Ma YC, Huang J, Ali S, Lowry W, Huang XY (2000) Src tyrosine kinase is a novel direct effector of G proteins. Cell 102: 635–646. 11007482 10.1016/s0092-8674(00)00086-6

[33] Baker MA, Hetherington L, Aitken RJ (2006) Identification of SRC as a key PKA-stimulated tyrosine kinase involved in the capacitation-associated hyperactivation of murine spermatozoa. J Cell Sci 119: 3182–3192. 16835269 10.1242/jcs.03055

[34] Heldin CH, Westermark B (1999) Mechanism of action and in vivo role of platelet-derived growth factor. Physiol Rev 79: 1283–1316. 10508235 10.1152/physrev.1999.79.4.1283

[35] Betsholtz C, Karlsson L, Lindahl P (2001) Developmental roles of platelet-derived growth factors. Bioessays 23: 494–507. 11385629 10.1002/bies.1069

[36] Asensio-López MC, Lax A, Pascual-Figal DA, Valdés M, Sánchez-Más J (2011) Metformin protects against doxorubicin-induced cardiotoxicity: involvement of the adiponection cardiac system. Free Radic Biol Med 51: 1861–1871. 21907790 10.1016/j.freeradbiomed.2011.08.015

[37] Asensio-López MC, Sánchez-Más J, Pascual-Figal DA, Abenza S, Pérez-Martínez, et al (2013) Involvement of ferritin heavy chain in the preventive effect of metformin against doxorubicin-induced cardiotoxicity. Free Radic Biol Med 57: 188–200. 23000260 10.1016/j.freeradbiomed.2012.09.009

[38] Asensio-López MC, Sánchez-Más J, Pascual-Figal DA, de Torre C, Valdes M, et al (2014) Ferritin heavy chain as main mediator of preventive effect of metformin against mitochondrial damage induced by doxorubicin in cardiomyocytes. Free Radic Biol Med 67: 19–29. 24231192 10.1016/j.freeradbiomed.2013.11.003

[39] Glickman MH, Ciechanover A (2002) The ubiquitin-proteasome proteolytic pathway: destruction for the sake of construction. Physiol Rev 82: 373–428. 11917093 10.1152/physrev.00027.2001

[40] Park H, Kaushik VK, Constant S, Prentki M, Przybytkowski E, et al (2002) Coordinate regulation of malonyl-CoA decarboxylase, sn-glycerol-3-phosphate acyltransferase, and acetyl-CoA carboxylase by AMP-activated protein kinase in rat tissues in response to exercise. J Biol Chem 277: 32571–32577. 12065578 10.1074/jbc.M201692200

[41] Bolster DR, Crozier SJ, Kimball SR, Jefferson LS (2002) AMP-activated protein kinase suppresses protein synthesis in rat skeletal muscle through down-regulated mammalian target of rapamycin (mTOR) signaling. J Biol Chem 277: 23977–23980. 11997383 10.1074/jbc.C200171200

[42] Nakashima K, Yakabe Y (2007) AMPK activation stimulates myofibrillar protein degradation and expression of atrophy-related ubiquitin ligases by increasing FOXO transcription factors in C2C12 myotubes. Biosci Biotechnol Biochem 71: 1650–1656. 17617726 10.1271/bbb.70057

