# Supplementary figures and images for: Metformin Protects Cardiomyocyte from Doxorubicin Induced Cytotoxicity through an AMP-Activated Protein Kinase Dependent Signaling Pathway: An In Vitro Study
Source: PLoS One. 2014 Aug 15;9(8):e104888. doi: 10.1371/journal.pone.0104888 (PMC4134245; doi:10.1371/journal.pone.0104888)

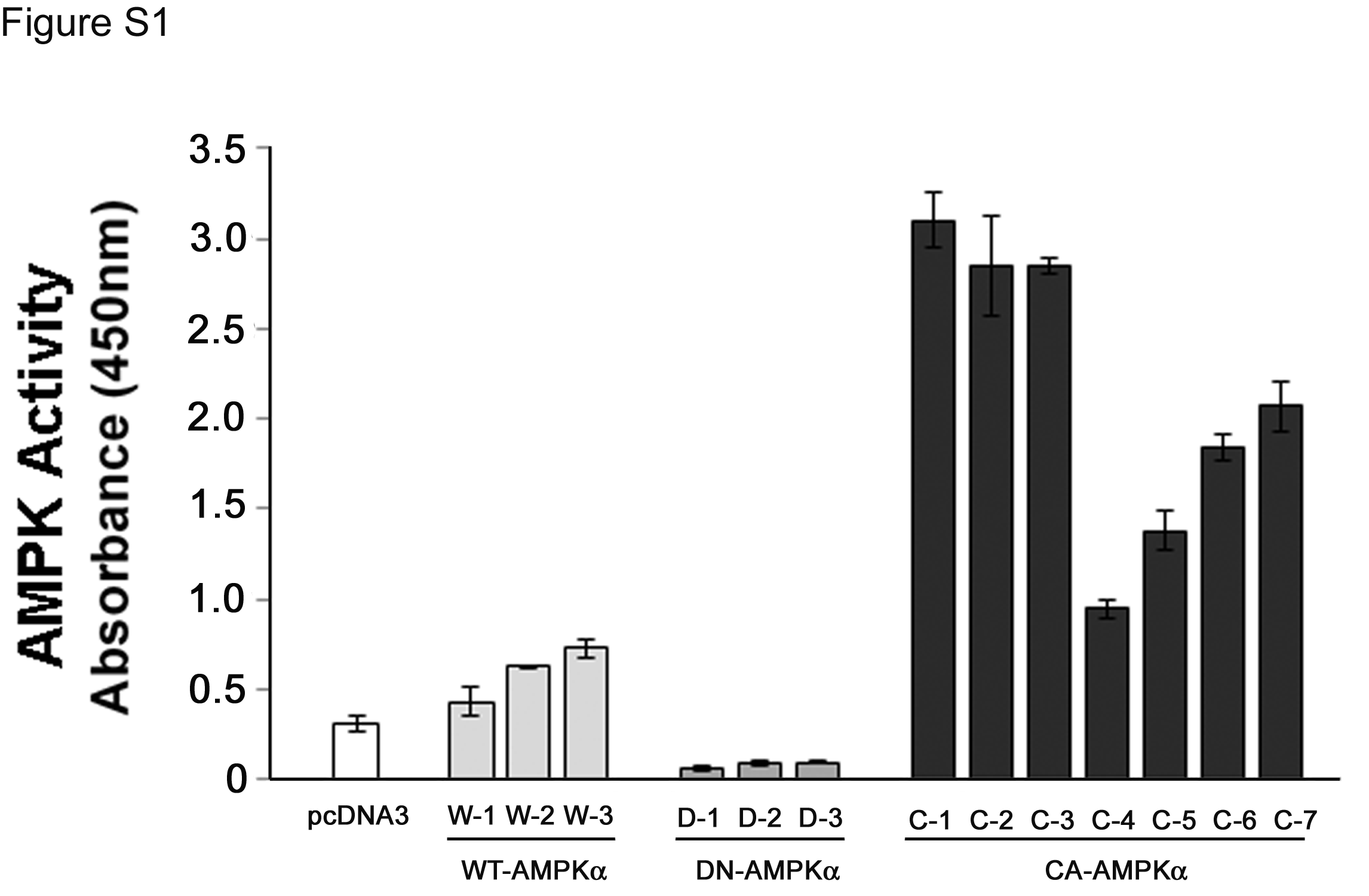

Supplement: Figure S1 — AMPK activities in empty vector (pcDNA3), wild type (WT), dominant negative (DN) or constitutively active (CA)-AMPKα transfected H9c2 cardiomyoblasts were as described in Materials and Methods. Three clones from each transfection were tested. Values represent mean ± S.D. (n = 4) from quadruplicate samples for each treatment. *, Significantly different from control (pcDNA3) (p<0.05). (TIF) [file pone.0104888.s001.tif]

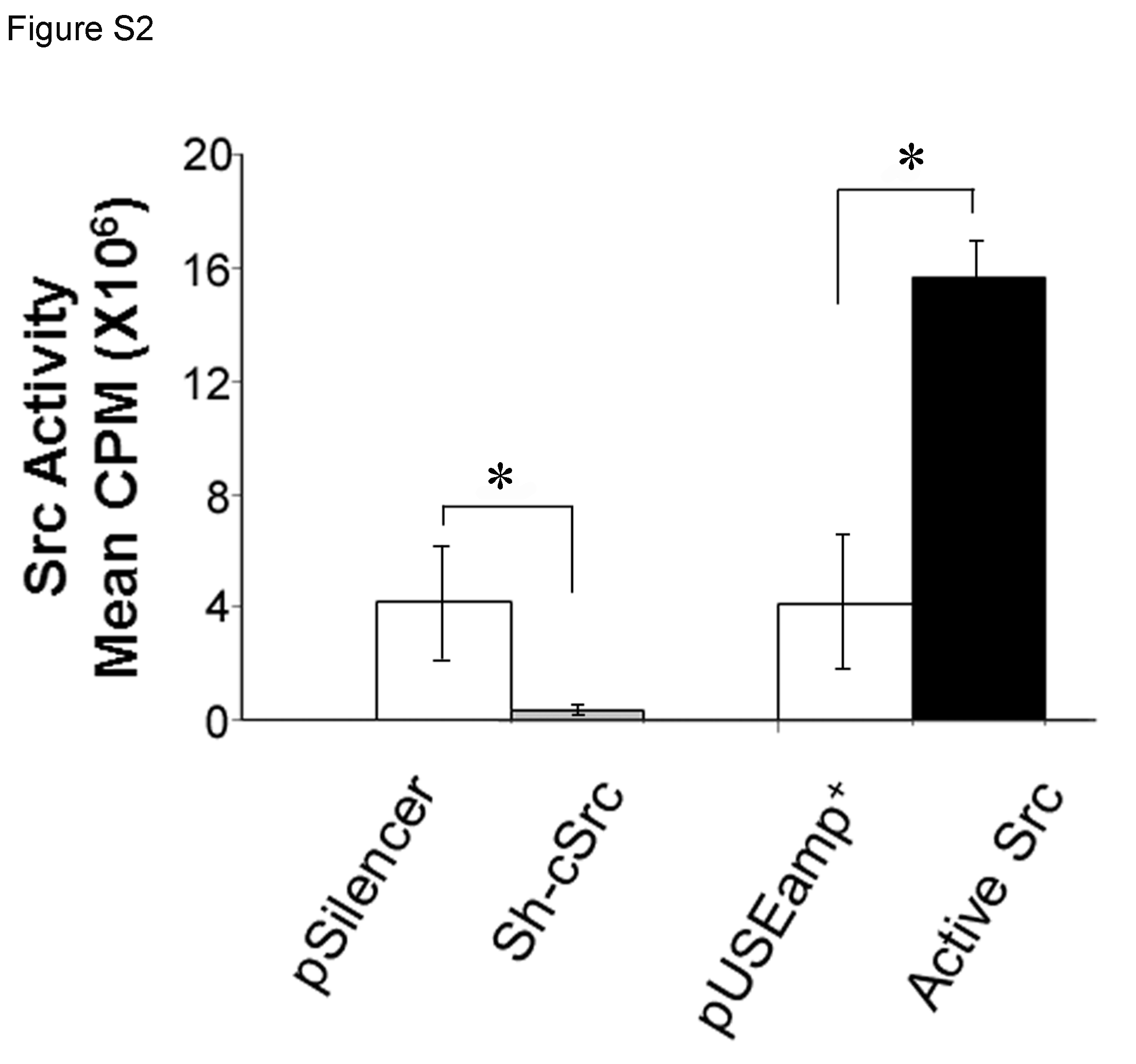

Supplement: Figure S2 — Src activities in H9c2 cells stably transfected with empty control vector, shRNA against cSrc (sh-cSrc) or constitutively active Src cDNA H9c2 cardiomyocytes were evaluated as describes in Materials and Methods. Values represent mean ± S.D. (n = 4) from quadruplicate samples for each treatment. *, Significantly different from control (pSilencer for sh-cSrc and pUSEamp- for active Src) (p<0.05). (TIF) [file pone.0104888.s002.tif]

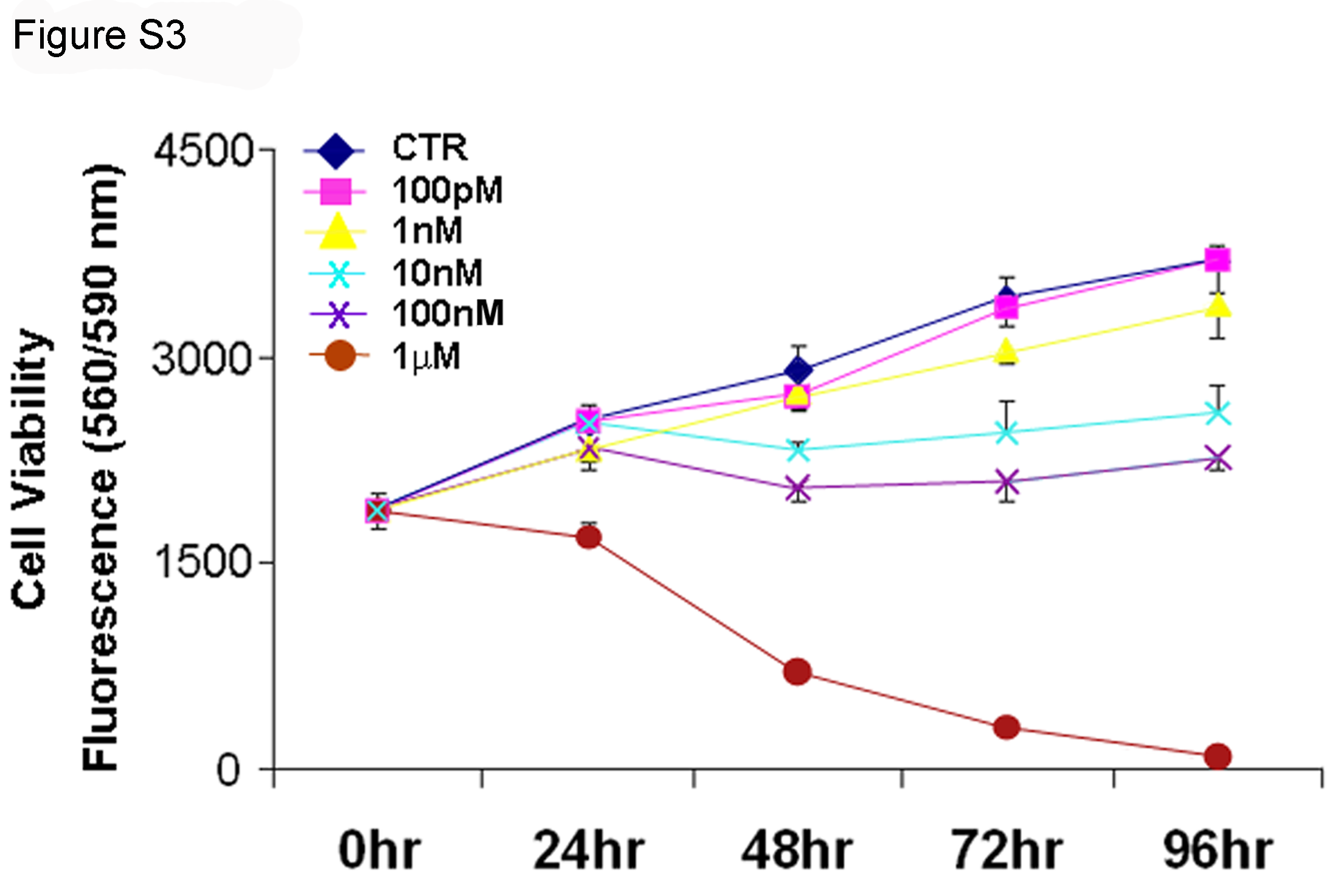

Supplement: Figure S3 — H9c2 cells were cultured for up to 96 hours with reduced FBS (1 with or without the indicated concentrations of Dox. Cell viabilities were evaluated as described. Values represent mean (n = 4) from quadruplicate samples for each treatment. (TIF) [file pone.0104888.s003.tif]

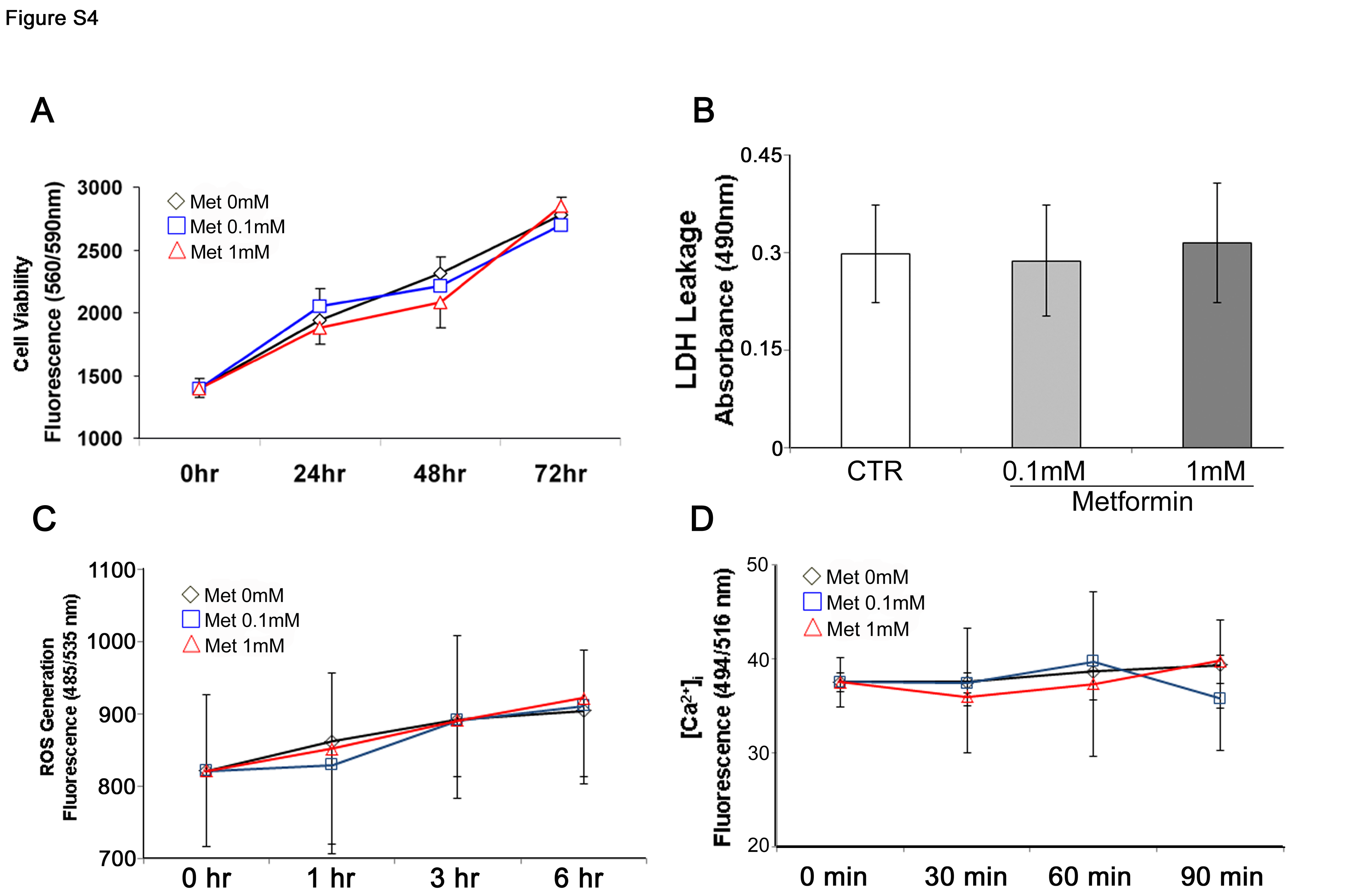

Supplement: Figure S4 — H9c2 cells were cultured for indicated durations with reduced FBS (1%) with or without the indicated concentrations of Met. Cell viabilities (A), LDA leakages (B), ROS generations (C) and [Ca2+]i (D) were evaluated as described. Values represent mean (n = 4) from quadruplicate samples for each treatment. (TIF) [file pone.0104888.s004.tif]

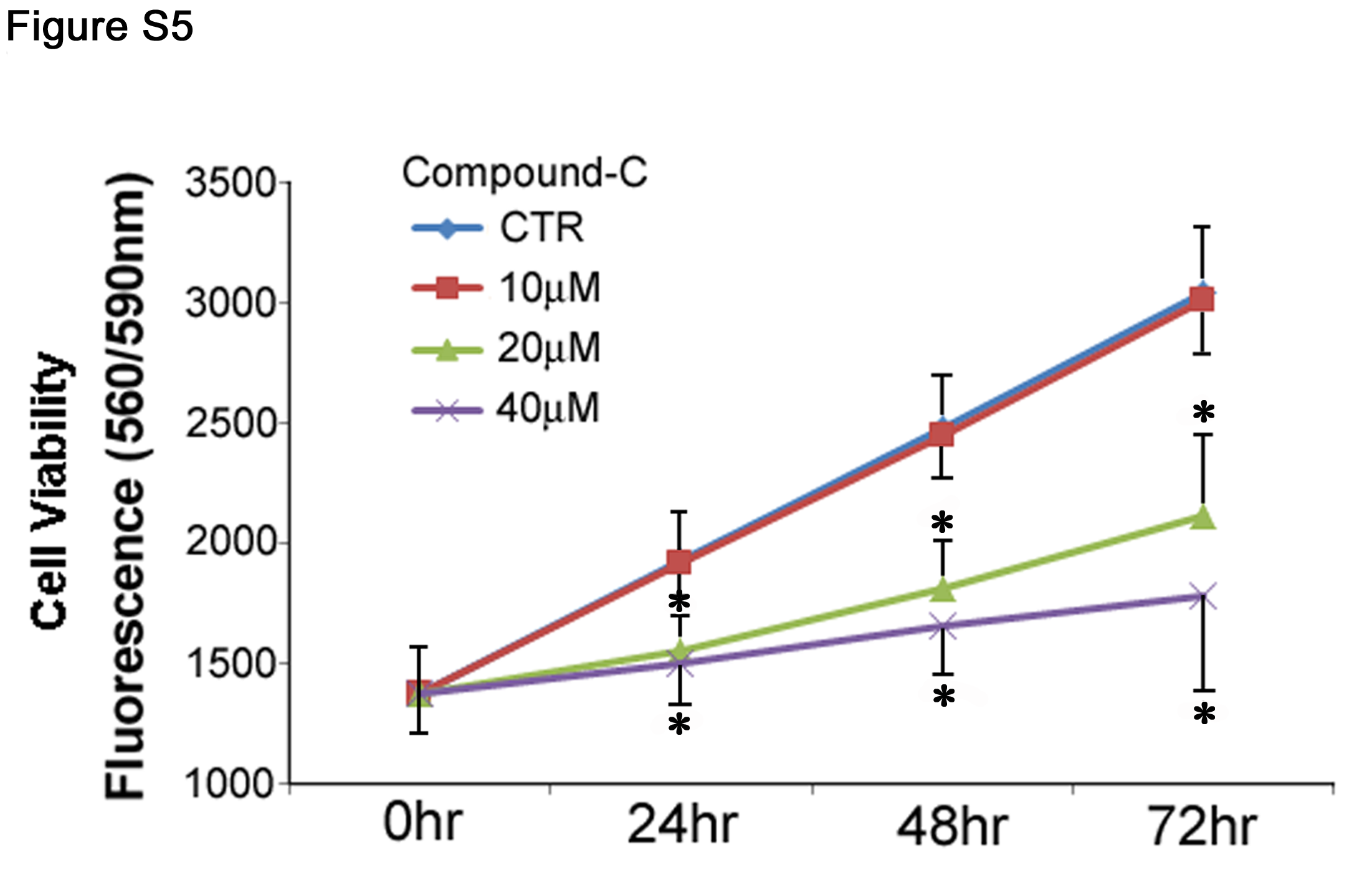

Supplement: Figure S5 — H9c2 cells were cultured for up to 72 hours with reduced FBS (1%) with or without the indicated concentrations of compound-C. Cell viabilities were evaluated as described. Values represent mean ± S.D. (n = 4) from quadruplicate samples for each treatment. *, Significantly different from control (CTR) (p<0.05). (TIF) [file pone.0104888.s005.tif]

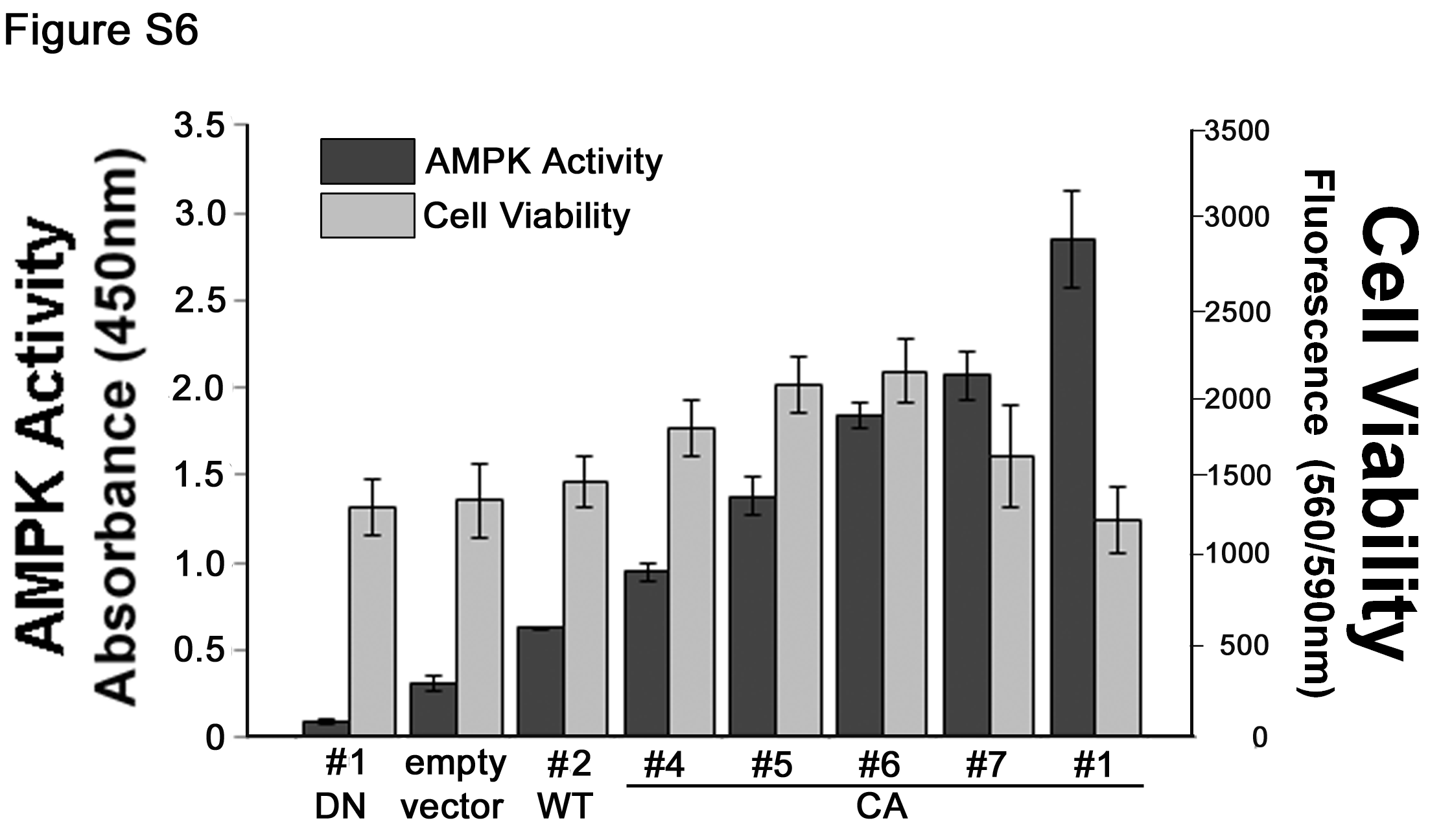

Supplement: Figure S6 — H9c2 cells which were stably transfected with the indicated plasmids were cultured for 72 hours with reduced FBS (1%) with 10 nM Dox and 0.1 mM of Met. Cell viabilities and AMPK activities were evaluated as described. (TIF) [file pone.0104888.s006.tif]

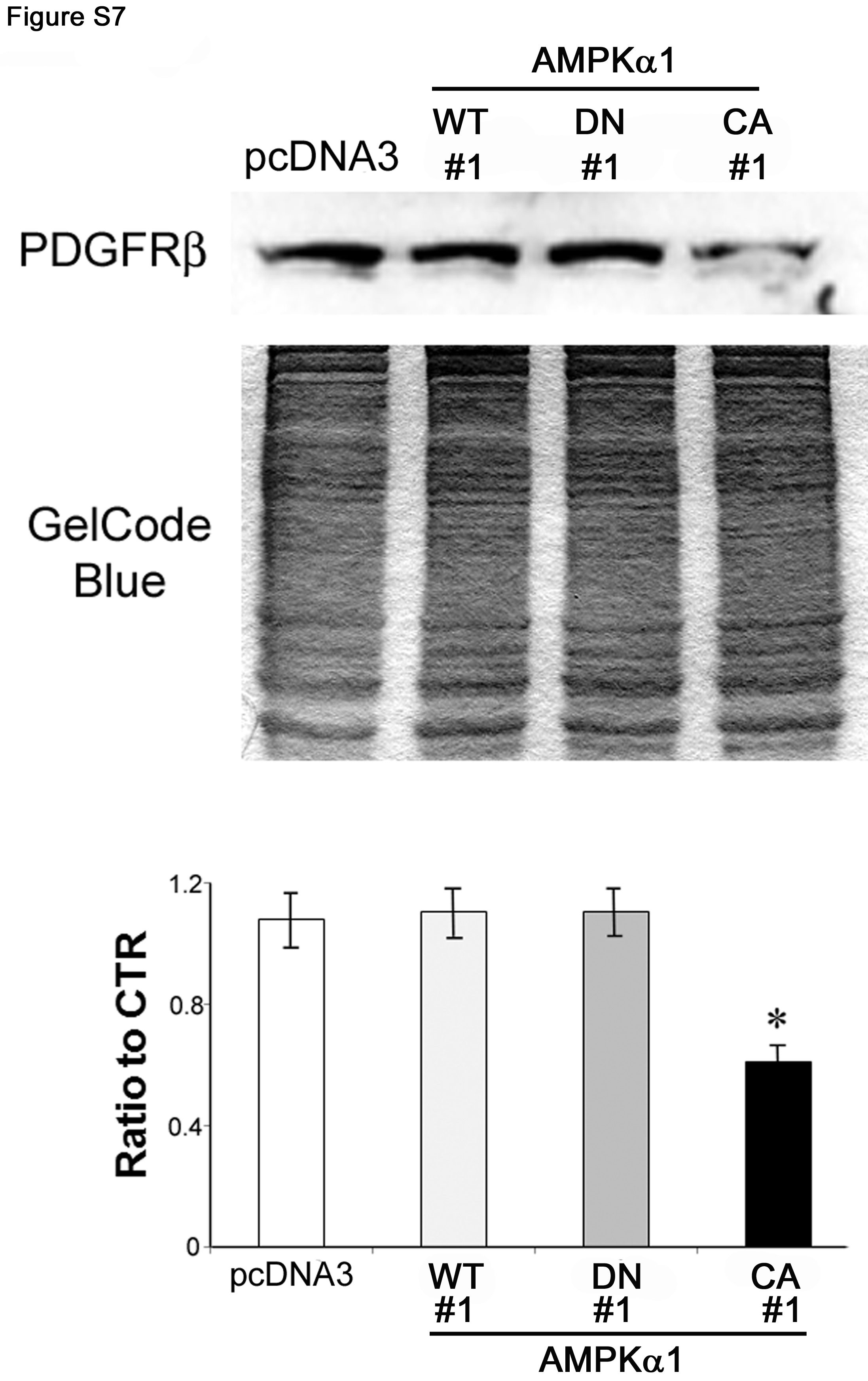

Supplement: Figure S7 — Cell lysates from quiescent AMPKα plasmids transfected H9c2 cells were subject to Western blotting using antibodies against platelet-derived growth factor receptor β-subunit (PDGFRβ). An image of the gel stained after transfer was shown as a loading monitor. The histogram shows the densitometric scanning results. *, Significantly different from control (pcDNA3) (p<0.05). (TIF) [file pone.0104888.s007.tif]
